# Meta‐analysis generates and prioritizes hypotheses for translational microbiome research

**DOI:** 10.1111/1751-7915.13047

**Published:** 2018-01-19

**Authors:** Claire Duvallet

**Affiliations:** ^1^ Department of Biological Engineering Massachusetts Institute of Technology Cambridge MA 02139 USA; ^2^ MIT Center for Microbiome Informatics and Therapeutics Cambridge MA 02139 USA

The potential for microbiome‐based therapeutics and diagnostics has generated significant excitement among scientists, clinicians and entrepreneurs. Much of basic microbiome research remains clinically relevant and readily translatable. Most human‐associated microbial communities are fairly non‐invasive to sample (e.g. stool, skin swabs, vaginal swabs), allowing for relatively easy access to clinical human samples. Once identified and developed, microbiome‐based interventions are likely to be non‐invasive and quickly adopted into standards of care. For example, identifying new uses for existing antibiotics and probiotics in microbiome‐related diseases could quickly transform clinical practice. From a clinical perspective, the microbial communities in our guts have been implicated in many diseases, including neurological conditions, metabolic disorders and autoimmune diseases. Encouraged by the remarkable success of faecal microbiota transplants (FMTs) in treating recurrent *Clostridium difficile* infection, clinicians are pursuing FMTs in hundreds of ongoing clinical trials. Finally, established biotechnology companies are expanding into the microbiome and start‐ups related to the microbiome are rapidly growing in number. Although the potential for impact is vast, successes have so far been limited: a recent clinical trial testing a defined microbial consortia for treating *C. diff* yielded disappointing results (Ratner, 2016), and results from FMT trials in conditions other than recurrent *C. diff* infection indicate that many conditions may have a variable and complex response to FMT (Moayyedi *et al*., 2015).

Despite the increasing research in this field, we have few generalizable insights into the human microbiome and disease, which may partially explain our current limited translational successes. Microbiome research has few established and standardized experimental and analysis methods, and individual studies often provide inconsistent or conflicting results. Clinical trials often report only outcome measures (e.g. remission rates) and broad microbial community characteristics (e.g. alpha diversity), without investigating specific microbiome alterations in detail. Microbiome studies which investigate specific microbial changes associated with disease frequently report finding ‘dysbiosis’, which is usually poorly defined and of uncertain therapeutic or diagnostic relevance to the disease of interest (Olesen and Alm, 2016; Hooks and O'Malley, 2017). Improving our foundational understanding of the relationship between the human microbiome and disease would likely lead to more and faster translational successes.

One way to generalize and integrate knowledge across studies is to perform a meta‐analysis of existing work (Glass, 1976). In the microbiome field, where basic science, clinical trials and commercialization are all happening simultaneously, meta‐analysis can be used to integrate the knowledge from these many perspectives and choose avenues to pursue which are more likely to lead to successful outcomes. For scientific researchers, meta‐analysis of existing studies increases our power to detect true signals, reduces the number of false positives by identifying consistencies across independent studies and can strengthen existing findings by demonstrating their reproducibility. In clinical trials and entrepreneurship, meta‐analysis can help identify therapies or products that are most likely to generalize across a variety of patient cohorts. Meta‐analysis across multiple diseases could perhaps even identify therapies that may be applicable to multiple indications.

## Meta‐analyses to better understand our tools

Early analyses that combined results from multiple microbiome studies did so to better understand the tools and methods used to process, analyse and interpret microbiome data. Some studies examined how different data processing methods impacted results and others compared the performance of different analytical techniques on various prediction tasks (Knights *et al*., 2011; Werner *et al*., 2011; Pasolli *et al*., 2016; Wang *et al*., 2016). These tasks were rarely motivated by a biological question but were rather used as benchmarks to evaluate the performance of different machine learning classifiers or analysis methods (e.g. statistical tests) on microbiome data. Another type of early meta‐analysis combined data sets to investigate the relative technical and biological contributions to variability in the microbiome (Lozupone *et al*., 2013). These authors found that the variation related to different microbial habitats (i.e. different body sites) is greater than the variation resulting from different studies (i.e. batch effects). However, it remains unclear how disease‐related variation compares to technical variation, and meta‐analyses which attempt to combine raw data from disparate studies should proceed with caution (Johnson *et al*., 2006; Sze and Schloss, 2016; Gibbons *et al*., 2017). In the future, methods‐focused meta‐analyses should be undertaken to identify ways to improve our tools, for example by developing methods to correct for batch across studies.

## Meta‐analyses to better understand relationships with disease

More recent meta‐analyses have combined insights from multiple data sets to investigate the generalizability and reproducibility of biological associations between human disease and the microbiome. These studies employ more traditional meta‐analytical techniques to synthesize findings from multiple studies with similar biological questions. The earliest of these focused on well‐studied conditions such as inflammatory bowel disease and obesity, and found few consistencies across multiple studies (Finucane *et al*., 2014; Walters *et al*., 2014; Sze and Schloss, 2016). Another approach has been to combine data sets to build generalized classifiers that could identify common features of a ‘healthy’ microbiome (Pasolli *et al*., 2016). In recent work, we extended these approaches to compare results across ten disease states, and found that there are consistent disease‐associated shifts and a shared response to disease (Duvallet *et al*., 2017). As the number of studies continues increasing, such meta‐analyses should continue to be undertaken to confirm and generalize biological insights across different patient cohorts and experimental designs.

Moving forward, meta‐analysis of microbiome studies could also be used to generate new hypotheses and motivate new microbiome‐based therapies. For example, in our study, we identified consistent disease‐associated patterns that suggested possible treatment strategies (Duvallet *et al*., 2017). We found that some diseases are consistently characterized by a depletion of commensals, which may therefore be suitable to probiotic interventions. On the other hand, diseases characterized by an overabundance of potentially pathogenic microbes may respond to narrow‐spectrum antimicrobial therapies. By comparing microbiome data across many diseases, we also found a set of bacteria which respond non‐specifically to disease, suggesting the potential for broad probiotic or antibiotic interventions that could work across a variety of conditions. Additionally, excluding these non‐specific microbes from follow‐up mechanistic studies or quests for diagnostic biomarkers in individual, diseases may help ensure that follow‐up findings are specific to the disease of interest.

Comparing results across diseases can also guide emerging research on conditions which have suspected links to the microbiome but little established research. Researchers can compare disease‐associated shifts found in these less‐studied conditions with those seen in diseases with established microbiome‐related aetiologies to identify shared mechanisms and potential successful therapies. In other words, if a disease which is newly being investigated exhibits similar patterns to diseases with established microbiome‐based interventions, then perhaps those interventions could work in this disease as well.

Finally, researchers could compare results from diseases with similar symptoms or aetiologies to identify microbiome alterations associated with more general physiological changes. For example, autoimmune disorders could be compared across multiple conditions and patient cohorts to better understand host–microbe immune interactions and to guide the development of general immune‐microbial therapies. Furthermore, comparing microbiome studies from neurological conditions may identify common mechanisms that could help us better understand the ‘gut‐brain’ axis and discover neuroactive microbial metabolites which could be used as targets for new treatments.

## Considerations for performing microbiome meta‐analyses

To perform a microbiome meta‐analysis, researchers must consider general meta‐analysis study design as well as certain challenges specific to microbiome data. As in any meta‐analysis, researchers must first decide which data sets to include and exclude. Many microbiome studies, however, contain information that can be used for additional analyses that were not included in the original publications. For example, Sze *et al*. used BMI metadata from non‐obesity studies to expand their obesity meta‐analysis (Sze and Schloss, 2016). In our meta‐analysis, we re‐purposed non‐*Clostridium difficile* diarrhoeal controls from the original Schubert *et al*. study as a separate case group for diarrhoea (Schubert *et al*., 2014; Duvallet *et al*., 2017). Researchers must decide whether and how to incorporate studies where the originally published analyses differ from but can be re‐purposed to fit the goals of the meta‐analysis. Additionally, confounders are common in microbiome research and patient cohorts are often quite heterogenous across studies (e.g. different inclusion and exclusion criteria and/or using healthy or non‐healthy controls; Morgan *et al*., 2012; Papa *et al*., 2012; Noguera‐Julian *et al*., 2016). Thus, researchers must ensure that enough data sets are being combined such that heterogeneity or confounded results do not drive the findings of the meta‐analysis (Glass, 1976).

In microbiome research, performing a traditional meta‐analysis by combining published *P*‐values is not feasible (Fisher, 1925; Glass, 1976). Studies often perform different and incomparable types of analyses (e.g. LEFSe (Segata *et al*., 2011), ‘enterotype’ analyses (Schubert *et al*., 2014), machine learning (Pasolli *et al*., 2016), etc.) which do not always provide readily available and comparable *P*‐values. Even if comparable *P*‐values were available, matching the identities of bacteria across studies can also be difficult. Different research groups use different databases and tools for taxonomic assignment, which often contain conflicting or non‐overlapping taxonomies.

Many microbiome studies now provide their raw sequencing data in public repositories, enabling meta‐analyses where raw data are re‐processed and directly compared. However, data reporting and deposition are not standardized, and many data sets may have incomplete or missing metadata (e.g. disease labels, replicate numbers, sample types). Additionally, issues related to privacy in microbiome data have been raised and not resolved (Franzosa *et al*., 2015). Thus, there are no standards for distributing raw microbiome data, and some data sets are publicly available, whereas others require significant approvals to access. Researchers performing microbiome meta‐analyses must consider whether to include controlled‐access data sets (e.g. data deposited in dbGaP or LifeLines‐DEEP) or restrict themselves to publicly available data alone.

Although performing meta‐analyses from raw data is ideal, it also comes with challenges. DNA extraction, amplification and sequencing methods all contribute to non‐biological artefacts, resulting in significant batch effects between studies. Researchers can attempt to correct for these batch effects computationally, but such methods may be effective in only a limited number of cases (e.g. case–control studies; Gibbons *et al*., 2017). Another option is to process each data set separately and compare results across studies. However, comparing results across studies requires reference‐based approaches, which limit the possible taxonomic resolution of results (Duvallet *et al*., 2017).

## Meta‐analysis to narrow and prioritize hypotheses

The field of microbiome research is expanding rapidly and raw microbiome data tend to be readily available, providing ample opportunity for high‐quality meta‐analyses. Entrepreneurs, clinicians and researchers all benefit from contextualizing their individual studies within the corpus of existing work. Disease‐associated microbiome signals that are consistently identified across many studies are less likely to be spurious results related to individual patient cohorts or confounders, and more likely to be truly associated with the disease of interest. Many microbiome‐based companies are being established, but early pioneers make it clear that we still have a ways to go before basic microbiome research can be successfully and robustly translated into targeted engineered therapies beyond FMTs.

Meta‐analysis can contribute to microbiome research and development efforts by narrowing and prioritizing hypotheses without requiring significant additional investment beyond supporting bioinformatics personnel and infrastructure. Companies working to develop robust therapies can leverage the existing knowledge and data in the field to pursue targets with higher likelihoods of success, which are those that rely on consistent patterns across studies. Data to perform such meta‐analyses are by definition already collected, allowing it to be integrated into many parts of the microbiome research and development pipeline without incurring many additional costs. Specifically, meta‐analysis is a crucial tool in early explorations before clinical trials are pursued. For example, commercializing a successful product or therapy requires efficacy across a variety of patients, and meta‐analysis can be used to computationally test hypotheses across a variety of patient cohorts without embarking on expensive clinical trials. As opportunities in the microbiome expand, so does the demand for bioinformaticians to analyse and interpret the increasing amount of data generated by preliminary investigations and clinical trials. Meta‐analysis is becoming one important part of the analytical and computational toolbox used by bioinformaticians to support and inform the translation of preliminary hypotheses into commercializable products.

## Conflict of interest

None declared.
